# Transfer Printing of Epitaxial Organic Semiconductor Films

**DOI:** 10.1021/acsami.5c25355

**Published:** 2026-02-16

**Authors:** Alessandro Minotto, Luisa Raimondo, Ilaria Lameri, Jacopo Perego, Angiolina Comotti, Angelo Monguzzi, Francesco Meinardi, Adele Sassella

**Affiliations:** Department of Materials Science, 9305 University of Milano-Bicocca, Milan 20125, Italy

**Keywords:** Transfer printing, organic semiconductors, epitaxy, rubrene, triplet fusion, singlet fission, thin films

## Abstract

Thanks to the commercial success of organic light-emitting diodes, organic electronics is now much more than just a niche alternative to traditional electronics. However, other types of devices based on organic semiconductors (OSCs) are still far from market readiness. A key limitation is that, in thin-film form, OSCs exhibit a high level of structural disorder. Of all strategies for growing films of OSCs, those relying on organic epitaxy yield films whose properties most closely resemble those of single crystals. Yet, this comes at a cost: conventional substrates for epitaxial growth are incompatible with practical device integration. To overcome this issue, we introduce a transfer printing method capable of relocating epitaxially grown films of OSCs from their native substrates to target, device-compatible ones. We demonstrate the feasibility of this approach by transferring highly crystalline rubrene films—grown via organic molecular beam epitaxy and characterized by coherently oriented, micrometer-scale domains and single-crystal-like optical response—from amino acid single crystals to technologically relevant substrates. Notably, morphology, optical characteristics, and photoluminescence dynamics of the films are fully retained following transfer.

## Graphical abstract

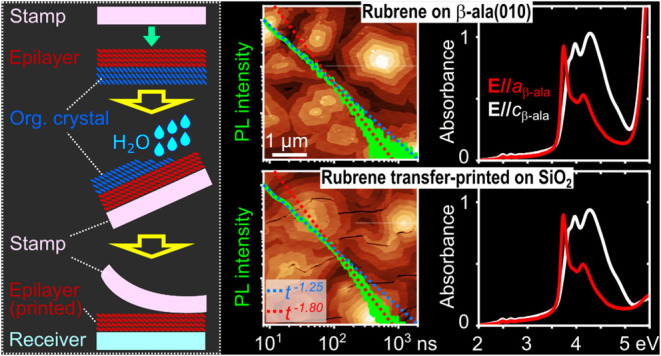

## Introduction

The degree of crystallinity in semiconductors plays a fundamental role in shaping their physical properties.^1^ In the case of organic semiconductors (OSCs), controlling the crystalline quality can be a complicated task. This is primarily due to the weakness of the interaction holding together molecules of different sizes and levels of rigidity. The resulting orientational and conformational degrees of freedom lead to structural disorder and polymorphism in solids of OSCs, particularly in thin films, where growth conditions, film thickness, and substrate properties can strongly influence the film morphology and structure. These aspects have an intrinsic influence on semiconductor properties, chiefly charge carrier mobility.^2,3^

It is thus no coincidence that significant advances in organic electronics have occurred alongside major strides in film structure engineering.^4,5^ However, devices based on OSCs consist of multilayered architectures with a heterogeneous composition, featuring interfaces between extremely dissimilar materials (e.g., organic/inorganic interfaces). Adhesion issues often occur, resulting in defects that can act as trap sites for charges and excitons. Yet, besides the requirement for high crystallinity at interfaces, controlling the orientation of molecules relative to an adjacent layer is frequently necessary.^6–8^ This is particularly important for devices relying on charge/exciton transport across crystalline layers. Electronic and optoelectronic properties of single-crystal OSCs—including charge mobility and exciton diffusion—are indeed highly anisotropic,^9,10^ and a substantial anisotropy can be observed also in polycrystalline films.^11–13^

From an interface-engineering perspective, epitaxial growth techniques yield the highest crystalline quality.^14–17^ The greatest control over film crystallinity and orientation is obtained when growth occurs on suitably chosen organic crystalline substrates that enable organic epitaxy, establishing registry between the molecular-scale corrugations of the substrate surface and the overlayer.^18^ This level of control is most effectively achieved using organic molecular beam epitaxy (OMBE) as thin film growth method,^19^ which provides exceptional interface purity—afforded by high- to ultrahigh-vacuum operation—and fine-tuning of growth parameters such as deposition rate and substrate temperature. These features are optimal to promote layer-by-layer growth of uniform, oriented crystalline films, even at very low thicknesses.^14^ However, these benefits—afforded by organic epitaxy through OMBE—are often offset by the technological inertness of organic crystalline substrates. Namely, integrating them into multilayered device architectures poses significant challenges in terms of design and fabrication.

One strategy to decouple the properties of the final architecture from those of the native substrates is to adopt layer-transfer techniques,^20–25^ offering the potential to unlock the production of high-efficiency organic electronic and photonic devices.

In this article, we present a broadly applicable method for transferring crystalline, highly oriented organic semiconductor films—grown by OMBE—from their original substrate to a different one. This proof of principle methodology reinterprets existing transfer-printing (TP) protocols^20–25^ by harnessing the water solubility of single crystals that are frequently used as substrates for organic epitaxial growth.^26–31^ We demonstrate its effectiveness using a model system—highly crystalline orthorhombic rubrene (RUB) films—grown by OMBE on (010)-oriented orthorhombic β-alanine (β-ala) substrates^14,15^ —which we successfully transfer onto technologically relevant substrates such as silica (SiO_2_) and Si(100). Remarkably, by atomic force microscopy, X-ray diffraction, optical spectroscopy, and photoluminescence analysis, we show that the film structure, optical response, and exciton dynamics are preserved after transfer.

## Results and Discussion

The method we propose, as illustrated in Figure 1, is effectively a revised version of the conventional TP using an elastomeric stamp made of polydimethylsiloxane (PDMS).^20–25^

**Figure 1:**
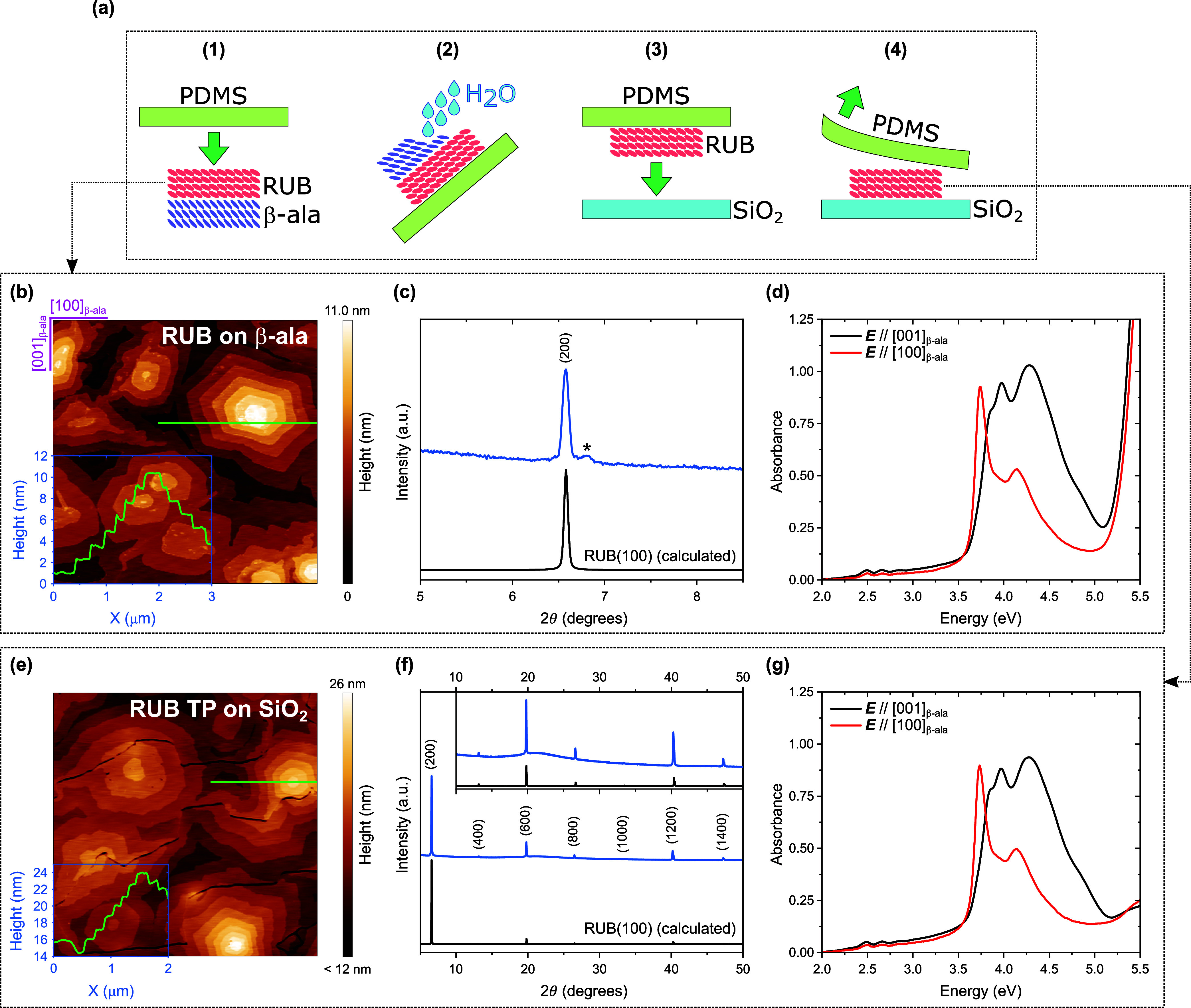
(a) Schematic of the transfer printing (TP) protocol. (b) (5 × 5) μm^2^ AFM height image of a 50 nm-thick RUB film on (010)-oriented β-ala and (c) corresponding XRD pattern. (d) Normal incidence optical absorption spectra, collected with linearly polarized light with ***E*** parallel to [100]_β‑ala_ (red curve) and parallel to [001]_β‑ala_ (black curve), of a 20 nm RUB film on its native β-ala substrate. (e) (5 × 5) μm^2^ AFM height image and (f) XRD pattern collected from the same RUB film in (b, c) after transfer printing (TP) from the native β-ala substrate onto SiO_2_. (g) Normal incidence optical absorption spectra of the film in (d) after TP onto SiO_2_. Insets in (b, e) show the height signal profiles measured along the green lines. The asterisk in (c) indicates a satellite peak at 6.8° 2θ, originating from an epitaxial native RUB oxide layer.^50^ Inset in (f) shows an enlargement of the XRD pattern between 10° and 50° 2θ. A constant background was subtracted from the spectra in (d, g), where the light polarization notation is referenced to the original β-ala(010) substrate.

The epitaxial organic semiconductor film is first grown by OMBE on a water-soluble single-crystal substrate. After contacting the stamp against the film (step 1 in Figure 1a), the epitaxy-enabling substrate is dissolved via deionized water rinsing (step 2). Residual water is then removed from the inked PDMS stamp by a gentle nitrogen flux before bringing it into contact with the receiving substrate (step 3). The dry semiconductor film is finally transferred to the receiving substrate by slowly (∼ 1 mm s^–1^) peeling off the stamp (step 4).

The key difference here with respect to conventional TP is that, after contacting the stamp against the epitaxial organic semiconductor film, the latter is not peeled off from the substrate. We adopted instead a strategy similar to the one reported by Yim et al.,^21^ who used a water-soluble sacrificial interlayer to pick up the semiconductor film—a conjugated polymer film—from a substrate. This was achieved by exploiting the fact that OSCs are typically insoluble in water (unless the conjugated system is properly functionalized). In our case, however, no interlayers are needed, as the substrate is dissolved via water rinsing. Note that our approach allows deterministic transfer of the epitaxial film,^32^ i.e., the film can be placed and precisely oriented on a specific region of a larger-area receiving substrate by leveraging the transparency of PDMS in the visible spectrum and the habit of the single-crystal donor substrate. Furthermore, the transfer procedure shown in Figure 1a overcomes the limitations of previous methods, which relied solely on dissolving the original crystalline substrate in water to transfer epitaxially grown films.^15,33–35^ Namely, using the PDMS stamp as an “intermediate” substrate offers two crucial advantages. First, it avoids the use of resins to protect the organic semiconductor film from fracturing when the substrate dissolves upon immersion in or rinsing with water.^33^ Second, only the epilayer comes into contact with water. As such, any risk of water damage of the receiving substrate or other layers present in the device architecture is prevented, since dissolution of the native substrate occurs on PDMS (Step 2) before printing (Step 3).

To validate the feasibility of our method, the combination of RUB as the organic semiconductor and (010)-oriented β-ala single crystals as the water-soluble donor substrate was employed because, via organic epitaxy, this model system enables the formation of one of the most prominent examples of oriented organic semiconductor films: highly crystalline orthorhombic RUB layers with single-crystal-like properties.^14,15^ Epitaxially grown RUB films^14,15,17,36^ represent a promising solution with a view to closing the gap between the exceptional optoelectronic properties of RUB single crystals, that is >10 cm^2^ V^–1^ s^–1^ hole mobility, several-μm-large exciton diffusion length, and near-unit singlet fission and triplet–triplet fusion efficiency,^9,37–39^ and the inferior ones of RUB films deposited using conventional methods. This discrepancy, which represents a key bottleneck for the integration of RUB in devices, originates from the inability to control polymorphism, crystallinity, and orientation under nonepitaxial film growth. In RUB solids, poor crystallinity not only affects their optoelectronic properties, but also their photostability.^14,17,40–44^

For obtaining the oriented RUB films on β-ala, we followed an optimized two-step OMBE growth protocol (full details of the deposition protocol are reported in the Experimental Section).^14,15^ The results of the morphological, structural and optical analyses of the as-grown epitaxial RUB films on β-ala and the corresponding data obtained from the same films after TP on SiO_2_ are shown in Figure 1b–d and Figure 1e–g, respectively.

The AFM height image in Figure 1b, collected from a 50 nm RUB film on β-ala, reveals a surface morphology characterized by micrometer-wide, hexagonal, multilayered islands. The step height between layers, as can be extracted from the cross-sectional profile in Figure 1b, is 1.32 ± 0.16 nm, matching the spacing between the monomolecular layers enclosed between (200) planes of the orthorhombic RUB polymorph (*a* = 26.86 Å, *b* = 7.19 Å, *c* = 14.43 Å).^45^ This confirms that RUB grows epitaxially on β-ala(010), with (100)_RUB_ in contact with (010)_β‑ala_.^14^ This is corroborated by X-ray diffraction (XRD) analysis, which is sensitive to the properties of the entire sample. Figure 1c shows a sharp diffraction peak centered at 2θ = 6.58°, corresponding to a distance of 13.44 Å, in perfect agreement with the one calculated for the 200 reflection of orthorhombic RUB.^45^ Notably, the morphology of the film in Figure 1b (and in Figure S1, showing a (10 × 10) μm^2^ AFM height image of the same film) is reminiscent of the one of RUB films grown on RUB single-crystal surfaces, i.e., the ideal underlayer for seamless homoepitaxial growth, which were obtained at deposition rates that are at least five times slower than those used in the present case.^46^

The polarization optical spectroscopy analysis (Figure 1d) provides a further demonstration of the high crystallinity and orientation of RUB films grown on β-ala. The profiles of the absorption spectra of a 20 nm-thick RUB sample, measured on *a* ≈ 3 mm^2^ area with light linearly polarized along [001]_β‑ala_ and [100]_β‑ala_, match those previously reported for RUB films grown on β-ala(010).^14,15^ Namely, Figure 1d reveals a single-crystal-like optical response,^47^ which is particularly evident in the higher energy end of the spectra (3.5–4.5 eV range), where the characteristic anisotropic response of crystalline (100)-oriented orthorhombic RUB on β-ala(010) is detected.^14,15^ Furthermore, in both spectra in Figure 1d the lowest-energy vibronic progression (2–3 eV range) lacks the 0–0 component at about 2.35 eV. As previously reported,^14,48^ this vibrationless component is indeed suppressed at normal incidence in the case of (100)-oriented orthorhombic RUB, since the parent electronic transition is [100]_RUB_-polarized (i.e., the corresponding dipole moment is perpendicular to the substrate), while the remaining components of the absorption manifold are detected because of vibronic-induced depolarization. A zoom of the spectra in the 2–3 eV range is included in Figure S1.

Remarkably, as shown in Figure 1e, the morphology of the RUB film is essentially unaffected by the TP process. The TP film on SiO_2_ exhibits the same multilayered morphology of the pristine film on β-ala (Figure 1b), with the same step height between layers (1.33 ± 0.21 nm), thus proving that the out-of-plane orientation of the original film is conserved. This occurs even if the stamping process, as shown in Figure 1e, can give rise to cracks in the film morphology, with cracks defining domains that appear unevenly raised from the SiO_2_ substrate surface. However, some cracks are visible also in the as-grown films on β-ala, as can be seen in the (10 × 10) μm^2^ AFM image in Figure S1, with crack density increasing with film thickness. Furthermore, in this work the RUB films are transfer-printed using a manual procedure. It is therefore reasonable to expect a lower density of transfer-induced faults if the stamping of the film is carried out using state-of-the-art micropositioning setups.^32^ Another factor that needs to be counted in to evaluate the effectiveness of the method is the quality of the β-ala substrate, which influences the defect density in the film before transfer. Nevertheless, it is beyond the scope of this work to optimize the β-ala single crystals and the cleavage process to obtain the crystalline substrate for the epitaxial growth.

The effectiveness of the protocol is supported by XRD data obtained from oriented RUB films transferred onto SiO_2_ (Figure 1f) and Si(100) (Figure S2). Indeed, the XRD patterns display only the family of peaks associated with the *h*00 reflections of orthorhombic RUB, confirming the conservation of the out-of-plane orientation. Moreover, the peak width of the 200 reflection of RUB before and after TP is comparable, indicating that the overall thickness of the film is preserved (Figure S2).

As for the morphology and structure, also the highly anisotropic optical response—characteristic of (100)-oriented orthorhombic RUB—is retained after transfer, as it can be inferred from the absorption spectra of the TP RUB film on SiO_2_ shown in Figure 1g. Crucially, the printed film preserves the size (≈0.5 cm^2^) and the macroscopic shape of the dissolved (010)-oriented β-ala crystalline substrate—typically an irregular hexagon with two opposite sides parallel to the [001]_β‑ala_ axis, previously identified under crossed polarizers. The preservation of film geometry during transfer allows precise manual control over film orientation on the receiving substrate (see Figure S3), to retain the original substrate-based polarization reference, and to target nominally the same macroscopic region with the probing beam in both pre- and post-transfer measurements, thereby facilitating a meaningful comparative analysis.

Comparison with the spectra before TP (Figure 1d) reveals that line shape and anisotropy are preserved. As expected, in the UV region above 5 eV the absorption edge of the native β-ala substrate disappears after TP, confirming complete substrate dissolution. In addition, a mild drop in overall absorption intensity can be noticed, attributed to material loss during transfer; however, this loss remains below 10% across all films investigated. Supporting Figure S4 presents the same spectroscopic comparison for films of varying thickness, demonstrating the reliability and reproducibility of the TP protocol. The close matching between the spectra of the pristine RUB film on β-ala and the TP one indicates that, also on a macroscopic scale, the high crystallinity and orientation of the film are preserved.

Conservation of crystalline orientation is extremely encouraging for the integration of crystalline RUB films in devices, especially those relying on controlled molecular packing for efficient energy harvesting and charge/exciton transport.^6^ Equally important, our oriented RUB films exhibit a relatively good optical transparency in the visible, due to the weakness of the only absorption band in this range (at normal incidence, the characteristic vibronic progression of RUB in the 2–3 eV interval is only weakly allowed by vibrational distortions in (100)-oriented orthorhombic films, *vide infra*). Transparency in the visible is indeed a desirable feature for organic thin-film transistors,^49^ or virtually any kind of application where the combination of optimal charge transport properties of crystalline RUB^15,37,38^ and transparency may be needed.

It is also important to note that the optical response of TP films remains essentially unchanged even after storage in air for months, as shown in Figure S5. This is remarkable, considering that the TP process is carried out in air under ambient light conditions, it involves the use of water, and the above-mentioned presence of transfer-induced faults. Defects, together with amorphous regions, are indeed known to be the sites where oxidation occurs at a faster rate in RUB solids.^14,43,44^ RUB crystalline domains, instead, benefit from the formation of an epitaxial native oxide layer, which acts as a passivation layer against further oxidation.^50^

To assess the viability of the TP method for fabricating optoelectronic devices, its impact on excitonic processes should be considered. To this aim, we analyzed the effect of the TP protocol on the photoluminescence (PL) of the RUB films. As for the optical absorption, the intensity and the spectral shape of the PL of crystalline RUB vary depending on its orientation in relation to the excitation and collection geometry, since the radiative transition is polarized along [100]_RUB_.^48^ The PL intensity, however, is intrinsically limited by near-unit-efficient singlet fission,^39^ whereby radiative singlet excitons split into pairs of dark triplet excitons. For this reason, the PL quantum yield of crystalline RUB—including that of our epitaxial films—is limited to only a few percent,^51^ falling below the measurement uncertainty of most integrating-sphere setups. Over 90% of the photons contributing to such a weak PL signal arise from the conversion of dark triplets into radiative singlets through triplet–triplet fusion.^39^ As a consequence, given the extended triplet diffusion length (over 4 μm) and lifetime (up to 100 μs) in crystalline RUB,^9^ the PL of crystalline RUB is extremely sensitive to the introduction of defects that may act as trap sites for triplet excitons.^40,52^ Defects can therefore interfere with the diffusion of triplets, ultimately modifying the rate of triplet–triplet fusion events that control the PL dynamics. Furthermore, harvesting of triplets by traps can produce localized singlet excitons, whose radiative decay gives rise to anomalous PL spectra and dynamics.^39,53^

We address these aspects in Figure 2, which illustrates the effect of the TP process from β-ala to SiO_2_ on the PL of a 50 nm RUB crystalline film.

**Figure 2:**
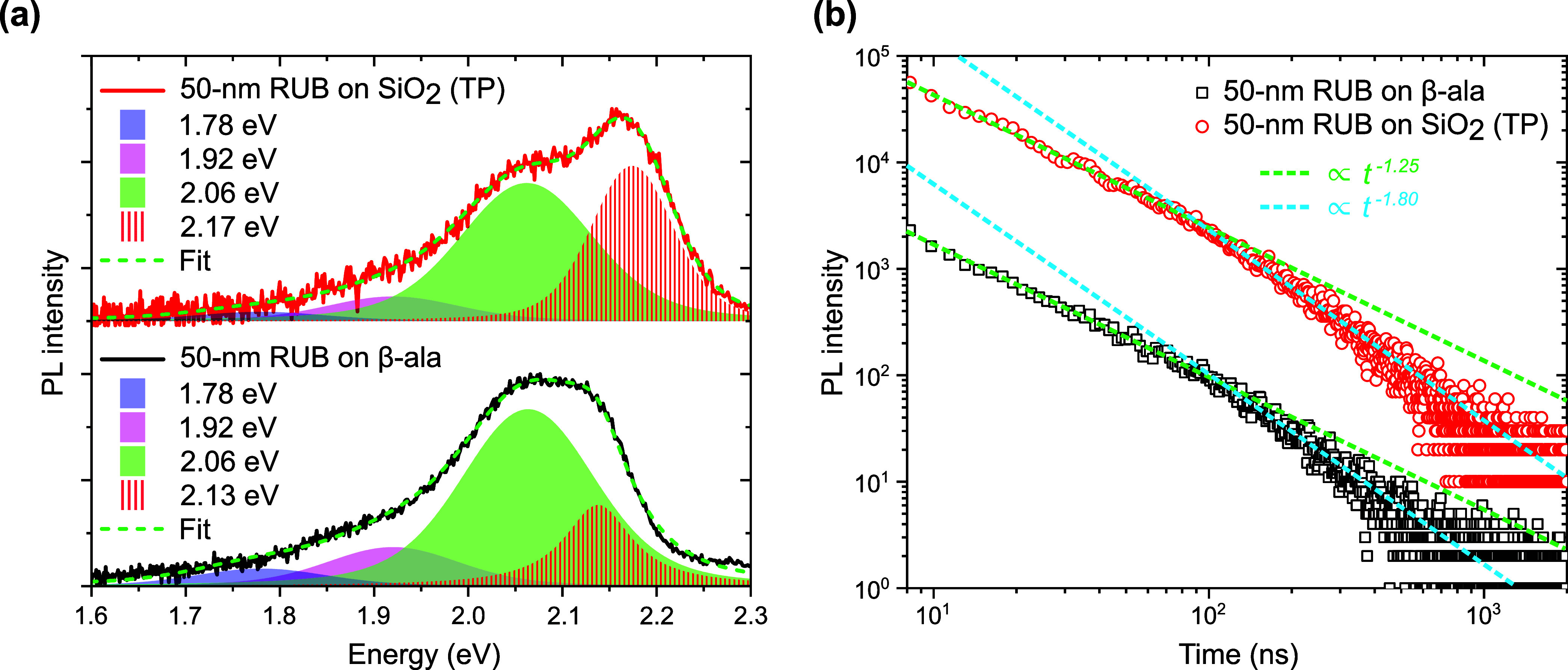
Steady-state PL (a) and transient PL (b) of a 50 nm RUB film on β-ala (black) and after TP on SiO_2_ (red) measured by exciting the samples at 2.49 eV (355 nm) with a 5 ns-pulsed laser. The shaded areas in (a) stand for the components of the Voigt multipeak fit (green dashed lines). The dashed lines in (b) correspond to the power-law functions that fit the transient PL data.

To compare the emission properties of the two samples—the 50 nm RUB film on β-ala(010) and the one on SiO_2_ after TP—we first examined the spectral shape of the PL by exciting it and capturing it at normal incidence with excitation energy of 3.49 eV (355 nm). The spectra collected from both samples are reported in Figure 2a. Note that we selected the normal excitation/collection geometry (i.e., parallel to the [100] direction of the coherently oriented RUB domains that make up the film) to avoid nontrivial corrections to the spectral profiles, accounting for the different composition, optical anisotropy, and thickness of the substrates. For both the as-grown and the TP film, the PL spectral shape is consistent with that reported for orthorhombic RUB single crystals.^48^ Specifically, both spectra can be fit by a sum of four Voigt profiles (represented by shaded areas in Figure 2a), corresponding to vibronic components characteristic of the electronic transition polarized along [100]_RUB_, which can be partly detected also in a direction parallel to [100]_RUB_ at sufficiently large solid angles, especially in the presence of scattering centers.^48^ In particular, for both films, the lower-energy vibronic replicas are centered at 1.78, 1.92, and 2.06 eV, in excellent agreement with the spectral positions reported in the literature for crystalline RUB.^48^ Furthermore, the corresponding Voigt profiles across the samples share the same full-width at half-maximum (FWHM) of about 0.17 eV, and the same ratio between the Gaussian and Lorentzian FWHM of about 2.5. Moreover, we observe no evidence of the frequently encountered alterations to the PL profiles, such as the anomalous PL peaking at 1.9 eV, which are typically associated with the presence of amorphous inclusions or photo-oxidation.^48^ This is particularly noteworthy for the TP sample, which reinforces the argument that the TP process has a negligible impact on the chemical integrity of the crystalline RUB films. This may be partly favored by the low defect density of the as-grown film,^44^ afforded by the optimized epitaxial growth. The only differences between the two samples are confined to the 0–0 vibronic band, whose peak center and FWHM were fitted independently and treated as free parameters. In the case of the β-ala sample (Figure 2a, black curve), the 0–0 peak is weaker than the 0–1 at 2.06 eV, red-shifted by ∼0.09 eV from the value expected from the literature (2.22 eV),^48^ and presents a FWHM of 0.09 eV, representing roughly a 2-fold narrowing compared to the lower energy vibronic features. This is likely due to [100]-polarized PL coupling into the ∼200 μm substrate and re-entering the film at oblique angles, where overlap with the 2.35 eV absorption band and consequent reabsorption effects become significant (Figure S5). By contrast, in the case of TP film (Figure 2a, red curve), the 0–0 component is the most intense one and only ∼0.06 eV red-shifted from the literature value, indicating reduced reabsorption and more efficient PL outcoupling, which reflects both substrate effects and, more importantly, the higher density of cracks in the TP film (Figure 1e), which scatter and thus enhance leakage of [100]-polarized PL. TP-induced changes in the molecular packing arrangement can instead be ruled out, as these would necessarily affect the lower-energy vibronic components as well. Furthermore, XRD and polarization optical spectroscopy (Figure 1) reveal no evidence of alternative polymorph formation or increased amorphous content in the TP film relative to the native one.

To provide further compelling evidence that the TP protocol proposed here does not affect exciton processes, we examined the PL decay dynamics. For the transient PL experiments, the samples were excited at 3.49 eV (355 nm) using a 5 ns-pulsed laser (the same one used to measure the PL spectra—see the Experimental Section). As known from the literature,^41,54^ the PL decay of RUB in the solid state is characterized by multiple components. One corresponds to the radiative deactivation of singlet excitons created by the pump pulse (also known as prompt emission, see Figure S6). These excitons have an associated lifetime of less than 15 ns, corresponding to the intrinsic singlet-exciton lifetime determined from transient PL measurements of dilute solutions or amorphous RUB films.^41^ In crystalline RUB, however, ultrafast singlet fission occurring on the picosecond time scale outcompetes both the radiative decay of RUB and, most importantly, the probability that singlet excitons encounter and interact with defects,^52^ such as those that may be introduced by the TP process. The most interesting components are instead the slower ones shown in Figure 2b, extending beyond 15 ns into the microsecond range. We mentioned earlier that, in RUB crystals, the delayed PL signal accounts for the majority of emitted photons, which originates from the recycling of dark triplets via triplet–triplet fusion. Consequently, the decay kinetics in this time window are highly sensitive to triplet exciton migration, as triplets are more likely than singlets to interact with or become trapped at defect sites—given their long diffusion length and lifetime in crystalline RUB (*vide infra*)—particularly those introduced by TP. Notably, the PL decays of the as-grown film (black squares in Figure 2b) and the TP one (red circles in Figure 2b), collected under the same excitation conditions, are essentially identical.

In quantitative terms, closer inspection of the log–log plots in Figure 2b reveals two distinct decay components in both samples, each following a power-law dependence, indicating that the delayed PL originates from the fusion of geminate triplet pairs generated via singlet fission.^52^ Up to about 100 ns, the exponent of the power law function which most accurately reproduces both sets of data (represented by the green dotted lines in Figure 2b) is approximately −1.25. It is known from the literature that the exponent of a power-law PL decay in OSCs depends on the anisotropy and dimensionality of exciton diffusion.^53,55^ Namely, a random walk model for the diffusion of the triplet excitons (between singlet exciton fission and geminate fusion) predicts an exponent of −1 for two-dimensional (2D) diffusion and −1.5 for one-dimensional (1D) and three-dimensional (3D) diffusion.^53,55^ However, exponents lying in between these values, as in our case, have been previously reported. For example, Bossanyi et al.^52^ observed a −1.3 exponent in films of RUB nanoparticles and ascribed it to a mixture of 2D and 3D triplet diffusion. Applying this interpretation to our case, the near-perfect match in decay kinetics suggests that the TP process preserves the balance between 2D and 3D triplet exciton diffusion in the film. The preservation of triplet diffusion anisotropy and dimensionality aligns with the XRD data in Figure 1c,1f, indicating that the orthorhombic structure of the coherently oriented crystalline domains and thickness of the film are not perturbed by TP.

The close correspondence between the PL dynamics of the as-grown and TP film persists even at longer time delays. Both decays exhibit a slope transition at around 100 ns, at which the power law changes from ∝*t* ^–1.25^ to ∝*t* ^–1.80^ (cyan dotted lines in Figure 2b). A previous work by Wolf and Biaggio^53^ showed that geminate fusion in RUB single crystals leads to a similar slope transition, with exponent changing from −1.18 to −1.66 at around 300 ns.^53^ This variation, in RUB single crystals, was attributed to a dimensionality crossover in triplet exciton diffusion from effectively 2D to 3D transport, i.e., from a regime in which triplet excitons migrate preferentially in the high-mobility (100) plane to a regime in which they also migrate along the least probable [100] hopping direction.^53^ In our epitaxial RUB films, the earlier onset (at ∼100 ns) of the second component and the steeper decay exponent (−1.8) indicate that additional processes may modify the exciton diffusion landscape compared with single crystals. Despite their high crystallinity, epitaxial RUB films inevitably exhibit a degree of static energetic and structural disorder, arising from grain boundaries between coherently oriented crystalline domains,^10^ particularly at the film surface. While the RUB single crystals in ref 53 exceed 100 μm along the [100]_RUB_ axis, our (100)-oriented RUB films are only tens of nanometers thick, making interactions between excitons and surface defects considerably more probable. Static disorder can localize triplet excitons and introduce trap-limited exciton diffusion pathways^56^ that can alter both the directionality and the rate of hopping. Namely, traps introduce intermediate states enabling exciton transfer between molecular stacks, promoting off-plane, trap-mediated hopping that partially relieves the intrinsic anisotropy of triplet motion.^10,57^ As a result, excitons can sample neighboring molecular planes earlier, producing an accelerated crossover from 2D to 3D diffusion. Concurrently, trapping can induce a departure from ideal random-walk behavior, giving rise to subdiffusive exciton transport.^10^ This interplay between trap-mediated interplane transfer and subdiffusion likely accounts for the faster decay observed in our RUB films relative to single crystals, and may also explain the deviation from the power-law trend at time delays beyond 1 μs.

In summary, despite the many processes at play, the as-grown and TP RUB films exhibit virtually identical decay kinetics over the entire time window from nanoseconds to microseconds, with matching power-law exponents and transition times. This invariance in exciton dynamics is consistent with the morphological, structural, and optical analyses. Together, these results demonstrate that the proposed TP protocol preserves the high crystallinity and orientation of the RUB film, as well as exciton transport and deactivation dynamics.

## Conclusions

To conclude, we have demonstrated, as a proof-of-principle, a transfer-printing strategy that is compatible with epitaxial crystalline organic semiconductor films grown by OMBE. This method leverages the water solubility of typical epitaxial substrates alongside an elastomeric stamp, previously employed for transferring both organic and inorganic films (including epitaxially grown inorganic semiconductors).^25^ This provides a general and versatile route to incorporate epitaxial organic semiconductors into functional device architectures, overcoming the constraints imposed by the native growth substrate. This is crucial for making the most of the precise control that epitaxy offers over crystallinity and molecular orientation, as these factors directly affect key processes in both light-emitting and light-harvesting devices, such as charge and energy transfer across interfaces.

Using RUB films epitaxially grown on β-alanine single crystals as a model system, we showed that the transfer preserves film morphology, crystallinity, optical response, and luminescence. By harnessing the typically poor solubility in water of organic semiconductors, we envisage that our transfer method could be used to transfer films of any organic semiconductor grown on water-soluble substrates. Arguably, the most critical aspect of the process is the use of water to dissolve the native substrate, which could in principle induce oxidation of the organic semiconductor. However, the conservation of both absorption and luminescence properties in the case of rubrene, which is known to be highly susceptible to oxidation by air and moisture,^43^ strongly reinforces our expectation that the methodology can be extended to other materials.

A key advantage of this approach is the deterministic positioning and precise control of crystalline film orientation on the acceptor surface, which is particularly valuable for enabling selective functionalization and integration with existing device architectures. Furthermore, the receiving substrate need not be limited to SiO_2_ or Si(100). The transfer process may be tailored to diverse substrates, including those with prefabricated features such as electrodes, nonplanar surfaces and mechanically flexible ones, through established adhesion-adjustment techniques. These strategies encompass pretreatment of the PDMS stamp via UV-ozone or oxygen plasma exposure,^23^ mild heating of the acceptor substrate during the delamination step to facilitate stamp release,^24^ or strategic exploitation of PDMS viscoelasticity to render the transfer kinetically favorable.^20^ Additionally, annealing of the inked PDMS stamp may be needed to minimize the risk of contamination of the target by water residues.

Finally, for RUB specifically, the synergy between the newly developed transfer method, the low-cost and easy-to-prepare β-alanine substrates, and the improved epitaxial growth protocol for highly crystalline, oriented films reported here establishes a scalable platform for integrating of RUB crystalline films into optoelectronic devices, which would benefit from the high hole mobility of crystalline RUB as well as its high singlet fission and triplet–triplet fusion efficiencies.

## Experimental Section

### Materials

Rubrene (RUB) powder was purchased from Acros Organics (99%). β-alanine (β-ala) was purchased from Sigma-Aldrich. Water-soluble single crystals of β-ala were grown following a previously reported protocol.^31^ The substrates for epitaxial growth—UV–visible-transparent, (010)-oriented β-ala crystalline sheets, with an area of ≈0.5 cm^2^—were obtained by cleavage of the β-ala single crystals, carried out in air just before film deposition.^14,15,31^

### Film Growth

The growth of RUB films was carried out on freshly cleaved β-ala(010) substrates via OMBE (base pressure ≈ 4 × 10^–10^ mbar) with a Knudsen-type effusion cell using a two-step protocol. The substrate temperature was set at 45 °C for the entire duration of the deposition. The nominal thickness of the film was monitored in situ using a quartz crystal microbalance. In the first deposition step, 1 nm thick RUB films were grown by setting the cell temperature to 180 °C, which affords a growth rate of ≈0.3 Å min^–1^. This rate, combined with the heating of the substrate, is necessary to obtain a uniform crystalline seed layer of RUB, covering the whole β-ala substrate.^14^ For the second step, the cell temperature was raised to 204 °C, for a growth rate of ≈3 Å min^–1^, to obtain films with final thickness up to 50 nm. The films were extracted immediately after the deposition end for characterization and transfer printing.

### Atomic Force Microscopy

AFM images were acquired in air using a Bruker Multimode Nanoscope V in intermittent contact mode with silicon tips (spring constant = 40 N m^–1^; resonance frequency ≈300 kHz; tip curvature radius <10 nm) and with an image resolution of 512 × 512 pixels.

### X-ray Diffraction

XRD patterns were measured in a Rigaku SmartLab SE powder diffractometer in Bragg–Brentano configuration, using Cu–Kα radiation. The tube was operated at 40 kV and 30 mA and the patterns were collected over a 2θ range of 5.0–50.0° with a step size of 0.01° and a scan speed of 0.3° min^–1^. The samples, grown on β-ala(010), or transfer-printed onto SiO_2_ or Si(100) substrates, were aligned along the *Z* directions and the ω_*x*_ and ω_*y*_ angles using the sample alignment procedure implemented in the software before the XRD measurement. The calculated pattern for orthorhombic rubrene was obtained from the crystal structure reported in literature (CCDC 605654).^45^ The preferred orientation of the sample was considered, and it was included in the calculation of the XRD pattern using the March-Dollase function.

### Polarization Optical Spectroscopy

Normal and oblique incidence optical transmission measurements were carried out in the 2.0–5.5 eV spectral range using a PerkinElmer Lambda 1050+ spectrometer, equipped with a depolarizer and Glan-Taylor calcite polarizers. The films were analyzed on a macroscopic scale, defined by a beam spot size of ≈3 mm^2^.

### Photoluminescence Characterization

For the PL experiments, samples were mounted and sealed in a cell in a N_2_-filled glovebox. Steady-state PL and transient PL were measured by exciting the sample with a 3.49 eV (355 nm) with a 5 ns-pulsed laser excitation (Laser Export, 1.5 mW, 10 kHz repetition rate). The steady-state PL was excited at normal incidence and collected in the same direction from the opposite side of the substrate with a TM-C10083CA Hamamatsu Mini Spectrometer. The transient PL measurements were carried out using a time-correlated single photon counting (TCSPC) setup. The laser beam excited the samples at non-normal incidence, while the PL was collected perpendicularly to the film surface. PL decays were measured by integrating the PL measured in the 550–650 nm range. The excitation intensity was kept below 1 μJ cm^–2^ to avoid alterations of the power-law PL decay by nongeminate bimolecular reactions in the investigated time window.

## Supplementary Material



## Notes

AFM height image of a 50 nm-thick RUB film on (010)-oriented β-ala; optical absorption spectra of a 20 nm crystalline RUB film on (010)-oriented β-ala and a 20 nm amorphous RUB film grown on amorphous SiO_2_ (Figure S1); XRD patterns of epitaxial RUB films on the native β-ala(010) substrate and after transfer printing onto Si(100) and amorphous SiO_2_ (Figure S2); normal incidence optical absorption spectra of a 20 nm TP RUB film with light polarization referenced to the edges of the receiving substrate (Figure S3); normal incidence optical absorption spectra of RUB films with thickness of 15, 20, and 25 nm on the native β-ala(010) substrate and on SiO_2_ (after transfer printing) (Figure S4); normal incidence optical absorption spectra of crystalline epitaxial RUB films right after transfer printing onto SiO_2_ and after 9 months of storage in air; optical absorption spectrum—collected at 45° incidence under p polarization—of a 50 nm crystalline epitaxial RUB after transfer printing onto SiO_2_ (Figure S5); transient PL of 50 nm-thick RUB film on (010)-oriented β-ala and instrument response function (Figure S6) (PDF)
