# Supplementary material for: Transfer Printing of Epitaxial Organic Semiconductor Films
Source: ACS Appl Mater Interfaces. 2026 Feb 16;18(7):11662–70. doi: 10.1021/acsami.5c25355 (PMC12954660; doi:10.1021/acsami.5c25355)
Supplement: Supplementary file 1 [file am5c25355_si_001.pdf]

Supporting Information

Transfer printing of epitaxial organic semiconductor films

Alessandro Minotto, Luisa Raimondo*, Ilaria Lameri, Jacopo Perego, Angiolina Comotti, Angelo Monguzzi, Francesco Meinardi, and Adele Sassella*

Department of Materials Science, University of Milano-Bicocca, Milan, Italy

Corresponding author's email: alessandro.minotto@unimib.it, luisa.raimondo@unimib.it

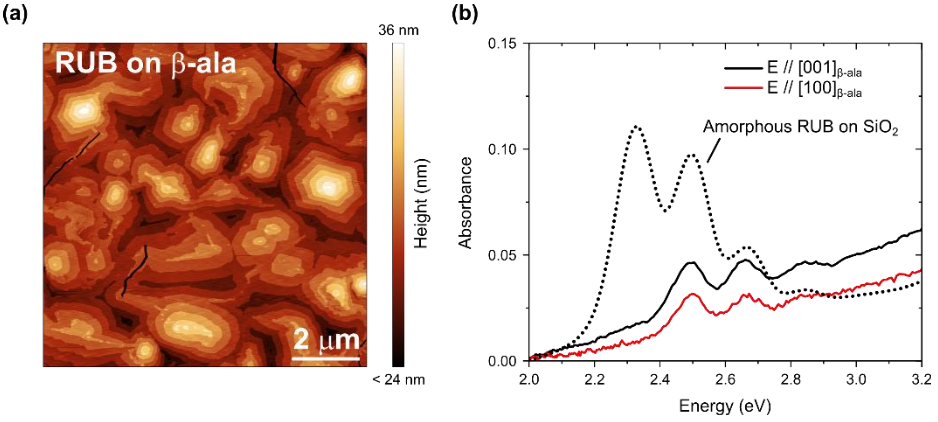

Figure S1. (a) (10×10) μm^2 AFM height image of a 50-nm-thick RUB film on (010)-oriented β-ala. (b) Normal incidence optical absorption spectra showing the vibronic progression corresponding to the lowest energy electronic transition of a 20-nm RUB film on its native β-ala(010) substrate (full lines) and of a 20-nm amorphous RUB film grown on an amorphous SiO_2 substrate (dotted line). The spectra of the crystalline film were collected with linearly polarized light with ***E*** parallel to $[100]_{\beta\text{-ala}}$ (red curve) and parallel to $[001]_{\beta\text{-ala}}$ (black curve). Note that 0-0 vibronic component at 2.35 eV is suppressed in the crystalline film, for the reasons discussed in the main manuscript. The light polarization notation is referenced to the native substrate, *i.e.*, the (010)-oriented β-ala crystal, previously aligned under crossed polarizers. A constant background was subtracted from the spectra.

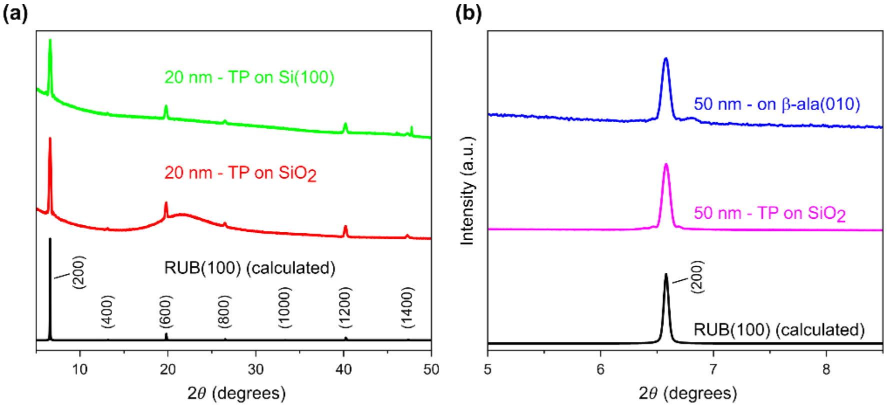

Figure S2. (a) XRD patterns collected from 20-nm crystalline RUB films after TP onto SiO_2 and Si(100). (b) Comparison between XRD patterns collected from crystalline 50-nm RUB films on the native β-ala(010) substrate and after TP onto SiO_2. An enlargement in the 5°- 9° 2θ range is shown to highlight the conservation of the peak width of the *200* reflection of RUB before and after TP.

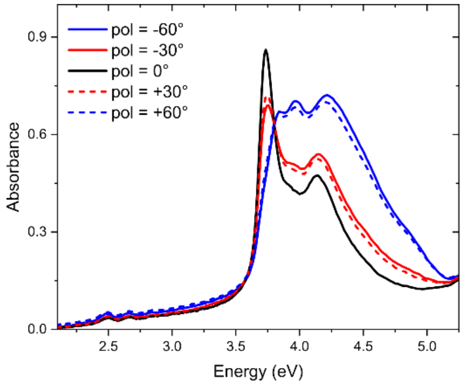

Figure S3. Normal incidence optical absorption spectra of a 20-nm-thick RUB film transfer-printed onto a square SiO_2 substrate, manually oriented so that two opposite sides of the hexagonally shaped native β-ala substrate – which are parallel to $[001]_{\beta\text{-ala}}$ and previously identified under crossed polarizers – align with a pair of edges of the receiving substrate. The black line corresponds to the spectrum collected with linearly polarized light with ***E*** parallel to the edges nominally aligned to $[100]_{\beta\text{-ala}}$. The remaining spectra were collected with linear polarization rotated by ±30° and ±60° with respect to the same receiving substrate edges. The virtually identical optical response at positive and negative rotations demonstrates that, although manual and requiring further optimization, the transfer-printing protocol reported here enables deterministic orientation of the films relative to receiving substrate features.

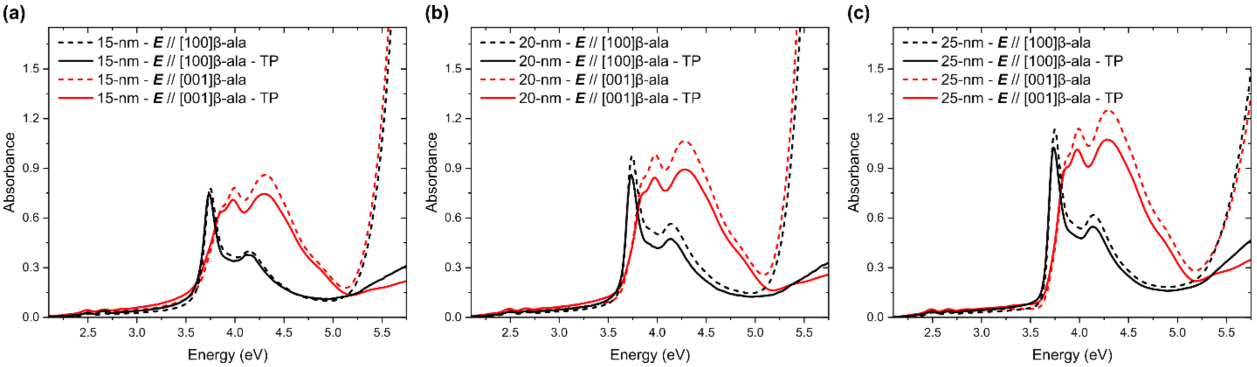

Figure S4. Normal incidence optical absorption spectra, collected with linearly polarized light with ***E*** parallel to $[100]_{\beta\text{-ala}}$ and parallel to $[001]_{\beta\text{-ala}}$, of RUB films with thickness of (a) 15-nm, (b) 20-nm and (c) 25-nm on β-ala(010) (dashed lines) and after transfer printing onto SiO_2 (full lines). The light polarization notation is referenced to the native β-ala(010) substrate. A constant background was subtracted from the spectra. The absorption intensity of the band peaking at 3.73 eV – detected at ***E*** // $[100]_{\beta\text{-ala}}$ – decreases by less than 10% after transfer, implying that material loss is limited, with potential for further improvement through protocol refinement. As discussed in the main manuscript and shown in Figure S2, the XRD results further support this conclusion, with the *200* reflection width remaining essentially unchanged after transfer. Note also that, for the systematic comparison of the absorption intensity, we focused on the 3.73 eV band because the higher energy transition – detected at ***E*** // $[001]_{\beta\text{-ala}}$ and peaking at 4.29 eV – lies closer to the absorption edge of β-ala. Consequently, for the ***E*** // $[001]_{\beta\text{-ala}}$ band, the difference in absorbance between the native and TP films is more significantly affected by the difference in refractive index dispersion between the two substrates (β-ala and SiO_2).

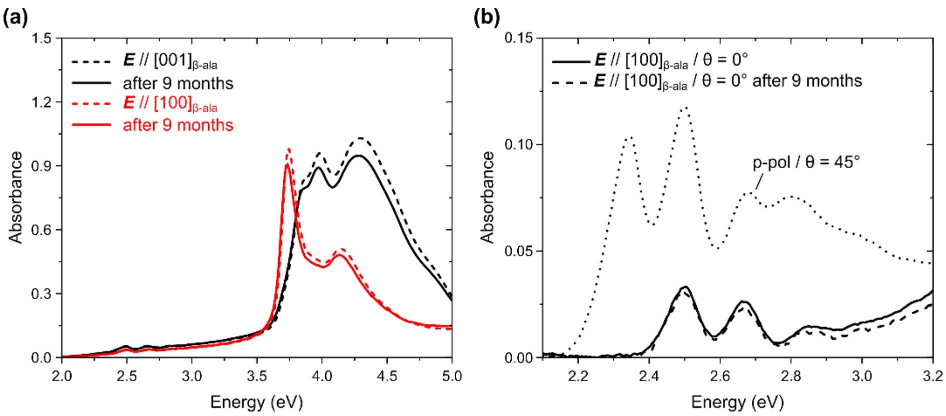

Figure S5. (a) Normal incidence optical absorption spectra, collected with linearly polarized light with ***E*** parallel to $[100]_{\beta\text{-ala}}$ (red curves) and parallel to $[001]_{\beta\text{-ala}}$ (black curves), of a 25-nm-thick RUB film right after transfer printing on SiO_2 (dashed lines) and after 9 months of storage in air (full lines). (b) Optical absorption spectra of a 50-nm-thick RUB film on SiO_2 (after transfer printing from the native β-ala substrate) collected with linearly polarized light. The full (dashed) line corresponds to the spectrum collected at normal incidence – with ***E*** parallel to $[100]_{\beta\text{-ala}}$ – right after TP (after 9 months of storage in air). The dotted line is the spectrum collected at 45° incidence – under p polarization – right after TP. The light polarization notation is referenced to the native substrate, *i.e.*, the (010)-oriented β-ala crystal, previously aligned under crossed polarizers. A constant background was subtracted from the spectra.

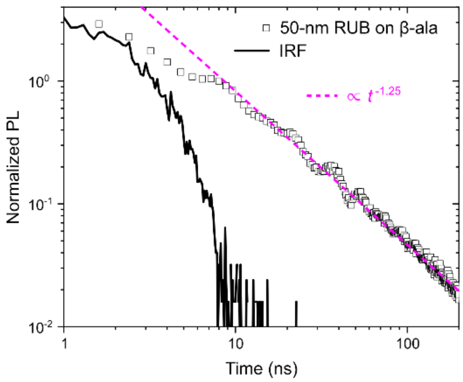

Figure S6. Transient PL (1-200 ns time window) of the 50-nm RUB film on β-ala (black squares) and instrument response function (IRF, black line). The purple dashed line corresponds to the power-law function fitting the experimental data.
